# Insulin-Like Growth Factor Binding Protein 6 in Rheumatoid Arthritis: A Possible Novel Chemotactic Factor?

**DOI:** 10.3389/fimmu.2017.00554

**Published:** 2017-05-18

**Authors:** Alessia Alunno, Onelia Bistoni, Mirko Manetti, Giacomo Cafaro, Valentina Valentini, Elena Bartoloni, Roberto Gerli, Arcangelo Liso

**Affiliations:** ^1^Rheumatology Unit, Department of Medicine, University of Perugia, Perugia, Italy; ^2^Department of Experimental and Clinical Medicine, Section of Anatomy and Histology, University of Florence, Florence, Italy; ^3^Department of Medicine and Surgery, University of Foggia, Foggia, Italy

**Keywords:** rheumatoid arthritis, IGFBP6, cell migration, synovial membrane

## Abstract

**Objectives:**

Immune cell migration from the bloodstream to target tissues is a hallmark of rheumatoid arthritis (RA) pathogenesis. The role of chemoattractants, mainly chemokines, and their possible targeting for therapeutic purposes have been under intense investigation over the last few years but the results were not as satisfactory as expected. The insulin-like growth factor binding protein 6 (IGFBP6), a direct inhibitor of insulin-like growth factor (IGF)-II, also exerts IGF-independent effects including tumor cell migration *in vitro*. We aimed to assess the expression of this protein in serum, synovial fluid, and synovial tissue (ST) of RA patients and to identify its possible chemotactic role in this disorder.

**Methods:**

IGFBP6 was measured in RA patients and healthy donors (HD) sera by Luminex xMAP^®^ technology and in ST of RA patients and osteoarthritis (OA) controls by immunofluorescence. The identification of circulating IGFBP6^+^ cells was evaluated by flow cytometry and an *in vitro* migration assay was arranged.

**Results:**

We demonstrated that IGFBP6 is able to induce greater *in vitro* migration of RA as compared to HD and OA T lymphocytes and is overexpressed in serum and ST of RA patients. This *in vitro* chemotactic activity can be partially inhibited by dexamethasone.

**Conclusion:**

Our findings suggest a pathogenic role of IGFBP6 in RA and support its possible targeting for therapeutic purposes.

## Introduction

Rheumatoid arthritis (RA) is a chronic inflammatory disorder that predominantly affects the joints. Immune cell recruitment from the bloodstream to the sites of inflammation is a crucial event in the development of synovitis. An excessive migration of these cells and their retention in the synovial environment, together with impaired apoptotic processes, chronically maintain the inflammatory response with the development of hyperplastic synovium and neoangiogenic phenomena. Rheumatoid synovial pannus can lead to damage of articular cartilage and bone with progressive joint destruction (1). Although chemokines and chemokine receptors, overexpressed in RA serum and synovial tissue (ST), appear to be leading players in orchestrating leukocyte recruitment to inflamed synovial membrane (2), the selective targeting of chemokines and their receptors has not yet led to the expected results in clinical trials (3, 4).

Cartilage and bone damage in RA may be considered the final result of catabolic mechanisms in the joint space exceeding anabolic pathways, similar to what occurs in tumor development and invasion. The predominating catabolic processes are thought to be cytokine-driven, upregulating proteases responsible for collagen and proteoglycan degradation. Since insulin-like growth factors (IGFs) are thought to be involved in the maintenance of articular cartilage and bone homeostasis, also in part through their ability to reverse the catabolic effects mediated by pro-inflammatory cytokines, a number of studies tried to analyze their role in rheumatoid synovitis. The results, however, were not conclusive.

In this setting, the regulation of IGF actions is critically important because of their essential physiological roles and the potential contribution of their dysregulation to a number of common diseases processes, including cancer and atherosclerosis. In fact, IGF activities are regulated by a family of six specific high-affinity binding proteins (IGFBPs) that are all able to inhibit IGF actions. Among the six IGFBPs, insulin-like growth factor binding protein 6 (IGFBP6) sparked increasing interest in recent years for its relative specificity in inhibiting IGF-II, whose circulating levels are several-fold higher than those of IGF-I in adult humans, thereby confirming its relevant physiological role, and for its ability to exert a number of IGF-independent activities. In particular, IGFBP6 appears to be able to enter the nucleus where it modulates cell differentiation and survival (5), to interfere with angiogenetic processes and, intriguingly, to promote cell migration. In particular, recent data suggest that it can induce tumor cell migration thereby worsening disease prognosis (6–9). On this basis, the investigation of IGFBP6 in RA may be of great interest in order to unmask a possible pathogenic role of this molecule in driving immune cell migration to target inflamed tissues.

Very recently, circulating antibodies reactive against citrullinated IGFBP6 have been described in RA patients (10). However, to the best of our knowledge, no data are available about serum concentration of IGFBP6 and its functional effects in RA. Moreover, only an outdated study unsuccessfully attempted to measure IGFBP6 in RA synovial fluid (SF), but it is to note that the assay employed to detect IGFBP6 in this investigation was scarcely sensitive (11). Therefore, the purpose of our study was to assess the expression of this protein with a highly sensitive assay in serum, SF, and ST of RA patients as well as to identify its possible chemotactic role in this disorder.

## Materials and Methods

### Study Cohort

Seventy patients with RA according to the 1987 American College of Rheumatology classification criteria were enrolled (12). Twenty-five sex- and age-matched healthy donors (HD) and 18 patients with osteoarthritis (OA) acted as normal and disease controls, respectively. RA patients were receiving stable biologic and/or conventional synthetic diseases modifying anti-rheumatic drugs (bDMARDs and csDMARDs respectively) over the 6 months prior to enrollment. The whole study was approved by the local ethic committee, and written informed consent was obtained in accordance with the Declaration of Helsinki.

### IGFBP6 Assessment in Serum and SF

Insulin-like growth factor binding protein 6 concentration was assessed in 34 RA and 12 HD serum samples and in 15 RA and 12 OA SF samples. Briefly, secreted IGFBP6 was measured using the MILLIPLEX MAP^®^ kit based on the Luminex xMAP^®^ technology (EMD Millipore Corp., St. Charles, MO, USA) according to the manufacturer’s protocol. The samples were incubated with the antibody-coupled microsphere, and later on with biotinylated detection antibody, then streptavidin–phycoerythrin (Pe) was added. The complexes were then scanned with a FLEXMAP3D reader (Luminex, Austin, TX, USA) set with 50 µl and 50 beads per bead set. All readings were performed by Bioclarma (Bioclarma srl, Torino, Italy).

### IGFBP6 Assessment in Peripheral Blood Mononuclear Cells (PBMCs)

Heparinized blood samples were obtained by five RA patients and five HD. PBMCs were isolated by density gradient, and surface staining was performed with fluorescein isothiocyanate, Pe or Pe-Cy7-labeled anti-human CD3, CD4, CD8, CD16, CD56, CD20, CD45RA, and CD45RO monoclonal antibodies and respective isotypes. After surface staining, cells were fixed with 4% paraformaldehyde and permeabilized with 0.1% saponin blocking buffer for subsequent intracellular staining with unconjugated rabbit anti-human IGFBP6 primary Ab and AlexaFluor647 donkey anti-rabbit secondary Ab. Up to four different fluorochromes were used in the same vial, and samples were analyzed using FACScalibur flow cytometer and CellQuestPro™ software (BD, San Jose, CA, USA).

### Synovial Specimens and Immunofluorescence Staining

Synovial biopsies were collected from seven patients with RA and six age- and gender-matched patients with OA who underwent surgical knee joint replacement. For routine histopathological analysis, paraffin-embedded synovial sections were deparaffinized and stained with hematoxylin and eosin. For immunofluorescence staining, tissue sections (5 µm thick) were boiled for 10 min in sodium citrate buffer (10 mM, pH 6.0), washed in phosphate-buffered saline (PBS), incubated in 2 mg/ml glycine for 10 min to quench autofluorescence caused by free aldehydes, and subsequently blocked for 1 h at room temperature with 1% bovine serum albumin (BSA) in PBS. The sections were then incubated overnight at 4°C with the following primary Abs diluted in PBS with 1% BSA: rabbit monoclonal anti-human CD3 (1:100 dilution; catalog number ab109531; Abcam, Cambridge, UK), mouse monoclonal Abs against human CD20 (1:200 dilution; catalog number M0755; Dako, Glostrup, Denmark), CD3 (1:10 dilution; catalog number ab17143; Abcam), CD14 (1:50 dilution; catalog number 347490; BD PharMingen, Heidelberg, Germany), CD31 (1:25 dilution; catalog number ab9498; Abcam), CD68 (1:50 dilution; catalog number M0876; Dako), vimentin (VIM) (1:50 dilution; catalog number M7020; Dako), and rabbit polyclonal anti-human IGFBP6 (1:50 dilution; catalog number ab135606; Abcam). The day after, the slides were washed three times in PBS and incubated for 45 min at room temperature in the dark with Rhodamine Red-X-conjugated goat anti-rabbit IgG or Alexa Fluor-488-conjugated goat anti-mouse IgG (Invitrogen, San Diego, CA, USA) diluted 1:200 in PBS with 1% BSA, as secondary Abs. Double immunofluorescence staining was performed by mixing mouse and rabbit primary Abs and subsequently mixing fluorochrome-conjugated secondary Abs. Irrelevant isotype-matched and concentration-matched mouse and rabbit IgG (Sigma-Aldrich, St. Louis, MO, USA) were used as negative controls. Nuclei were counterstained with 4′,6-diamidino-2-phenylindole (DAPI; Chemicon International, Temecula, CA, USA). Synovial sections were then mounted with an antifade mounting medium and examined with a Leica DM4000 B microscope (Leica Microsystems, Mannheim, Germany). Fluorescence images were captured with a Leica DFC310 FX 1.4-megapixel digital color camera equipped with the Leica software application suite LAS V3.8 (Leica Microsystems). Densitometric analysis of the intensity of immunofluorescent staining was performed on digitized images using the free-share ImageJ software (NIH, Bethesda, MD, USA; online at http://rsbweb.nih.gov/ij).

### Migration Assay

A total of 1 × 10^6^ PBMCs isolated as above from eight RA patients, four OA patients, and six HD were resuspended in 100 µl of complete medium with or without dexamethasone (dex) 5 × 10^−6^ M and seeded on the upper side of a 3 µm pore transwell. Six hundred microliters of either complete medium with recombinant IGFBP6 (1 µg/ml) or complete medium without the recombinant protein were put in the basolateral side of the transwell. After 2 h and 30 min incubation at 37°C, 5% CO_2_, the basolateral medium was harvested for counting of migrated cells and their phenotypic analysis by flow cytometry as detailed above. IGFBP6-induced cell migration was defined as the percentage of cells migrated in the medium with the protein normalized for the control (cell migrated in the medium without the protein).

### Statistical Analysis

Data were analyzed with SPSS 21.0 software using the Mann–Whitney *U* test and the Spearman’s correlation coefficient. *p* values <0.05 were considered significant.

## Results

### IGFBP6 Expression in RA Serum and SF

**Figure 1:**
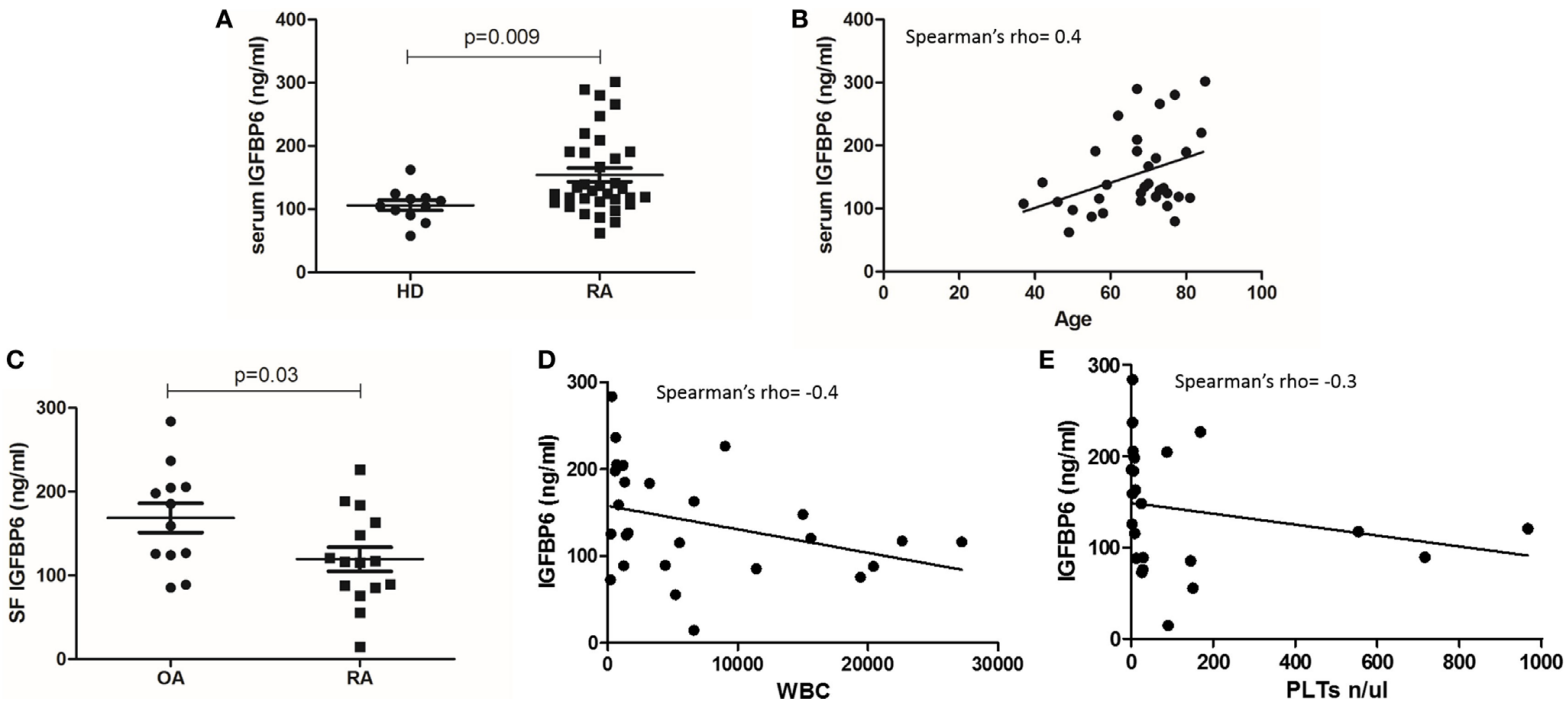
**Insulin-like growth factor binding protein 6 (IGFBP6) expression in serum (A) of rheumatoid arthritis (RA) patients and healthy donors (HD) and in synovial fluid (SF) of RA patients and osteoarthritis (OA) controls (C)**. IGFBP6 concentration is significantly higher in RA serum compared to HD and is significantly lower in RA SF compared to OA SF. Correlation between serum IGFBP6 and patients’ age **(B)**, SF IGFBP6 and SF white blood cell (WBC) count **(D)**, and SF platelet (PLT) count **(E)**. SF IGFBP6 concentration is inversely correlated to both SF WBC and PLT counts. *p* values shown in panels **(A,C)** were calculated with Mann–Whitney *U* test.

Table 1 summarizes the demographic and clinical features of RA patient cohort. We first sought to investigate the concentration of IGFBP6 in the serum of RA patients and we found that it was significantly increased compared to HD (Figure 1A). Intriguingly, IGFBP6 serum concentration was directly correlated with age in RA patients but not in HD (Figure 1B). In striking contrast, the concentration of IGFBP6 in RA SF was significantly lower compared to OA SF (Figure 1C). Moreover, when cumulating RA and OA patients, we observed an inverse relationship between IGFBP6 concentration and the number of both white blood cells (WBCs) and platelets (PLTs) in SF (Figures 1D,E).

**Table 1:** **Demographic, clinical, and serological features of RA patient cohort**.

Number of RA patients	70
Female gender	56 (80)
Age (years)^a^	57 ± 2.2
Age at diagnosis (years)^a^	48 ± 0.9
Disease duration (years)^a^	10 ± 2
Autoantibodies	
None	20 (29)
RF+/− anti-CCP+	50 (71)
Erosions	41 (58)
Nodules	4 (6)
Pulmonary involvement	6 (8)
csDMARDs	70 (100)
bDMARDs	38 (54)
DAS-28^a^	2.9 ± 1.2

*^a^Values are expressed as mean ± SEM. All other values are expressed as number of patients (%)*.

*csDMARDs, conventional synthetic diseases modifying anti-rheumatic drugs; bDMARDs, biologic diseases modifying anti-rheumatic drugs; DAS, disease activity score; RA, rheumatoid arthritis*.

### IGFBP6 Intracellular Expression in RA and HD Peripheral Blood

**Figure 2:**
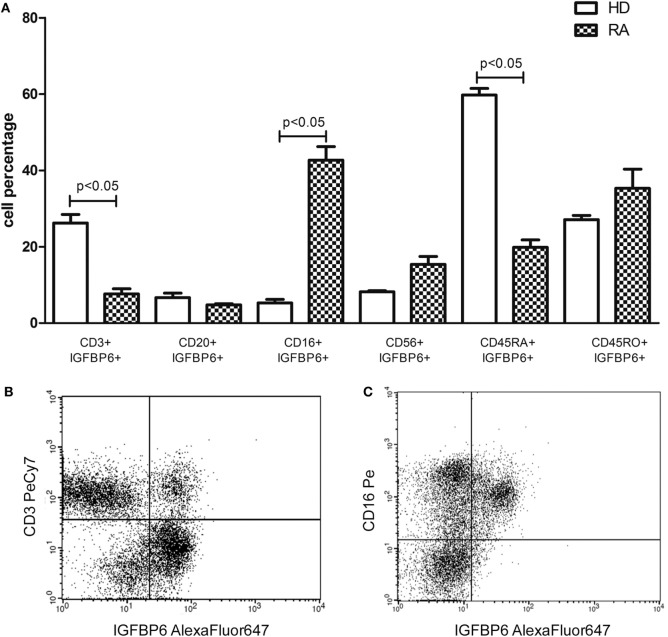
**Intracellular expression of insulin-like growth factor binding protein 6 (IGFBP6) in peripheral blood mononuclear cells (PBMCs) from rheumatoid arthritis (RA) patients and healthy donors (HD) (A) and representative flow cytometry dot plots (B,C) gated on total PBMCs of IGFBP6 expression in CD3^+^ cells and CD16^+^ cells in a patient with RA**. In RA PBMCs CD16^+^IGFBP6^+^ cell percentage is higher compared to HD while the percentage of CD3^+^IGFBP6^+^ cells is lower compared to HD. In addition, in HD the largest proportion of IGFBP6^+^ T cells coexpresses CD45RA. All *p* values were calculated with Mann–Whitney *U* test.

Subsequently, we assessed intracellular expression of IGFBP6 in circulating PBMCs from RA patients and HD. In HD, IGFBP6 was mainly expressed by PB CD3^+^ T lymphocytes and the evaluation of CD45 isoforms revealed that the vast majority of them were naïve T cells (CD45RA^+^CD45RO^−^) (Figure 2A). Conversely, in RA patients IGFBP6 was mainly detected in PB CD16^+^ monocytes and only a small proportion of CD3^+^ cells coexpressed IGFBP6 (Figure 2A). IGFBP6 was also detected in a small proportion of CD20^+^ B lymphocytes, but no differences could be observed between RA and HD. (Figures 2B,C) show two representative stainings for CD3 and CD16 in RA.

### IGFBP6 Expression in RA and OA ST

**Figure 3:**
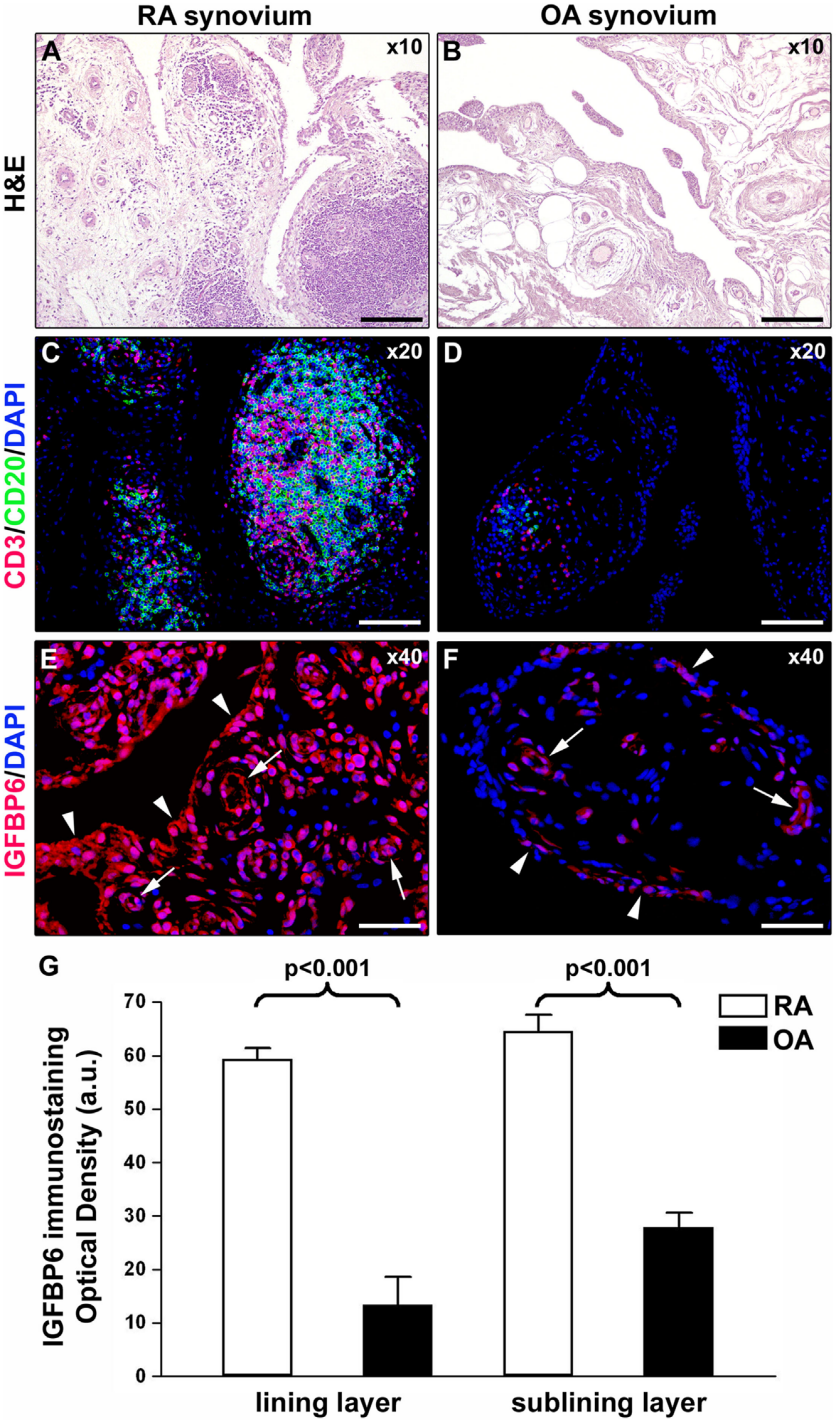
**Insulin-like growth factor binding protein 6 (IGFBP6) expression in synovial tissue from patients with rheumatoid arthritis (RA) and osteoarthritis (OA)**. Representative microphotographs of synovial membrane sections from patients with RA **(A,C,E)** and patients with OA **(B,D,F)** are shown. **(A,B)** Hematoxylin and eosin (H&E) staining. **(C,D)** Double immunofluorescence staining for CD3 (red) and CD20 (green) with 4′,6-diamidino-2-phenylindole (DAPI, blue) counterstain for nuclei. RA synovial membrane displays either diffuse immune cell infiltrates or ectopic lymphoid structures composed of T and B cells **(A,C)**. Few scattered T and B cells are present in OA synovium **(B,D)**. **(E,F)** Immunofluorescence staining for IGFBP6 (red) with DAPI (blue) counterstain. IGFBP6 is strongly expressed in different cells of the lining and sublining layers of RA synovium **(E)**. Faint expression of IGFBP6 is detected in OA synovium **(F)**. Arrowheads indicate synovial lining layer. Arrows indicate microvessels in synovial sublining layer. Original magnification: ×10 **(A,B)**, ×20 **(C,D)**, and ×40 **(E,F)**. Scale bar: 200 µm **(A,B)**, 100 µm **(C,D)**, and 50 µm **(E,F)**. **(G)** Densitometric analysis of IGFBP6 immunofluorescent staining intensity in lining and sublining layers of RA (*n* = 7) and OA (*n* = 6) synovium expressed as optical density in arbitrary units (a.u.). Data are mean ± SD. Student’s *t*-test was used for statistical analysis.

**Figure 4:**
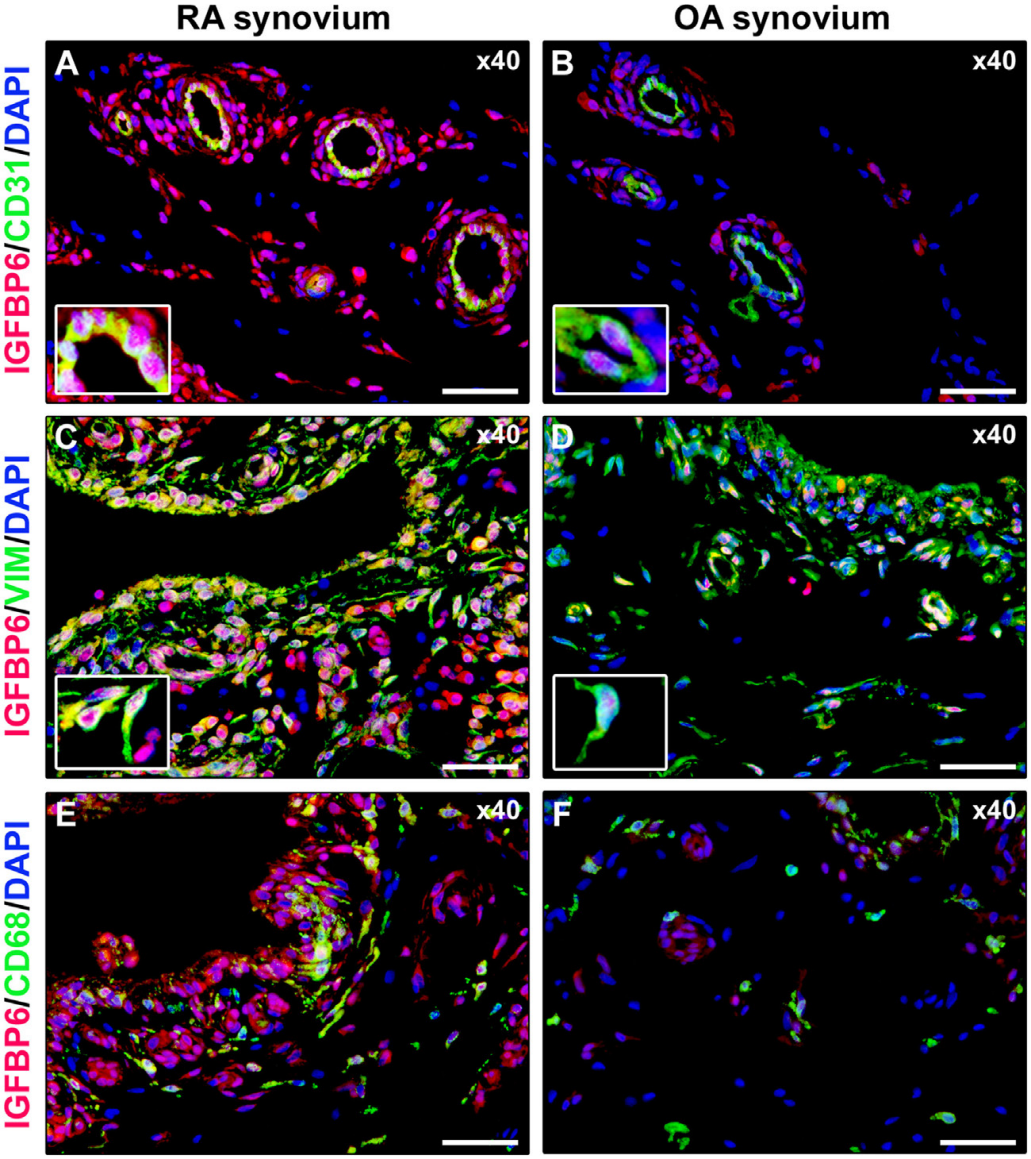
**Insulin-like growth factor binding protein 6 (IGFBP6) expression in different synovial resident cell types**. Representative microphotographs of synovial sections from rheumatoid arthritis (RA) patients **(A,C,E)** and osteoarthritis (OA) controls **(B,D,F)** subjected to double immunofluorescence staining for IGFBP6 (red) and the pan-endothelial cell marker CD31 (green) **(A,B)**, the fibroblast marker VIM (green) **(C,D)**, or the macrophage marker CD68 (green) **(E,F)**. Nuclei are counterstained with 4′,6-diamidino-2-phenylindole (DAPI; blue). In RA synovium, IGFBP6 is strongly expressed in CD31^+^ vascular endothelial cells **(A)**, VIM^+^ fibroblast-like synoviocytes (FLS) **(C)**, and CD68^+^ macrophages **(E)**. Weak IGFBP6 immunopositivity is detectable in the same cell types of OA synovium **(B,D,F)**. Insets: higher magnifications of double-immunopositive cells from the respective panels. Note the intense immunopositivity for IGFBP6 localized either in the cytoplasm or in the nucleus of RA synovial endothelial cells and FLS. Original magnification: ×40 **(A–F)**. Scale bar: 50 µm **(A–F)**.

**Figure 5:**
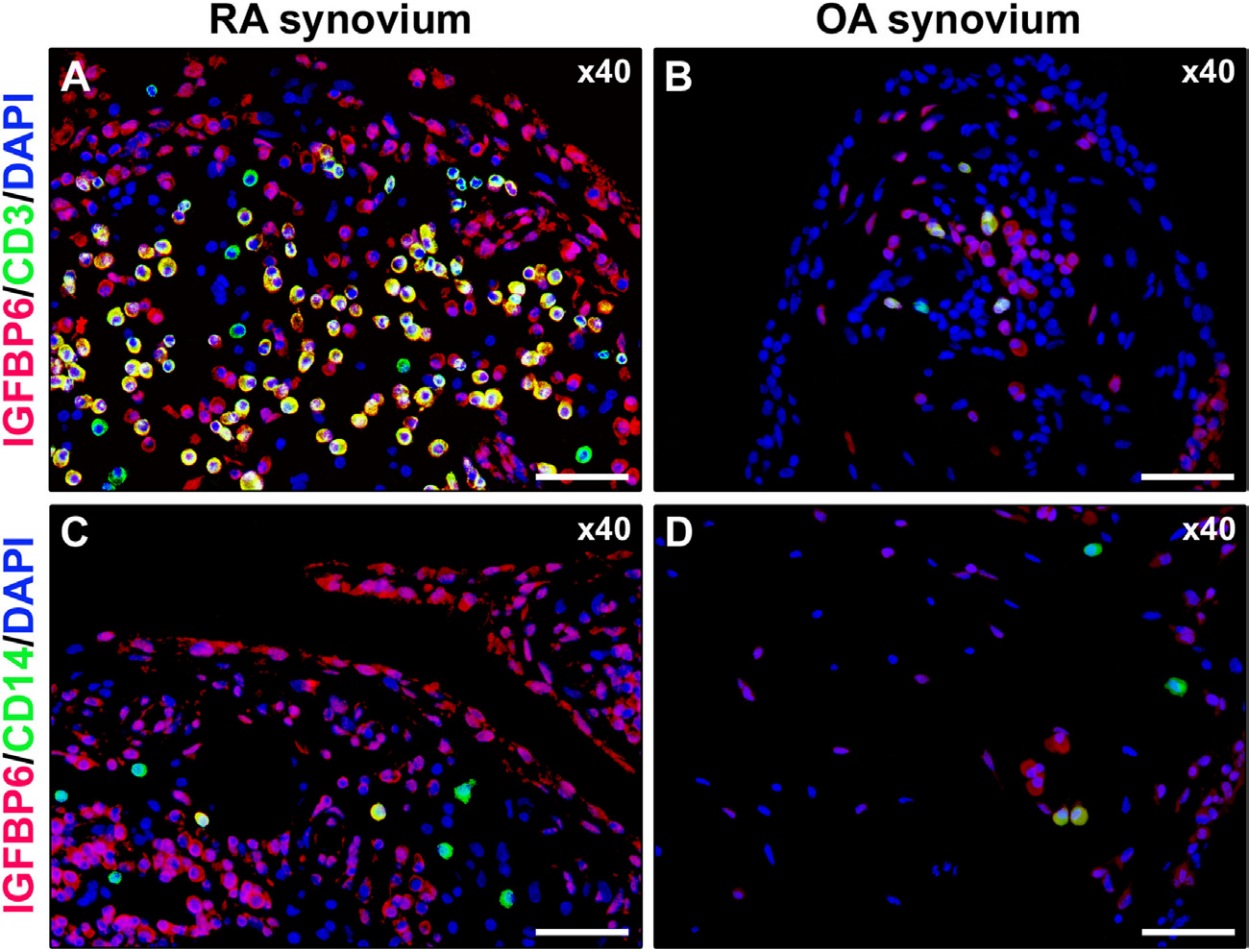
**Insulin-like growth factor binding protein 6 (IGFBP6) expression in synovial infiltrating immune cells**. Representative microphotographs of synovial sections from rheumatoid arthritis (RA) patients **(A,C)** and osteoarthritis (OA) controls **(B,D)** double immunostained for IGFBP6 (red) and the T-cell marker CD3 (green) **(A,B)** or the monocyte marker CD14 (green) **(C,D)**. Nuclei are counterstained with 4′,6-diamidino-2-phenylindole (DAPI; blue). Numerous CD3^+^ T cells displaying strong immunopositivity for IGFBP6 (yellow cytoplasmic staining) are present in RA synovial membrane **(A)**. Only a few CD14^+^ monocytes coexpressing IGFBP6 can be observed in RA synovium **(C)**. Original magnification: ×40 **(A–D)**. Scale bar: 50 µm **(A–D)**.

As displayed in Figures 3A–D, either diffuse immune cell infiltrates or ectopic lymphoid structures composed of CD3^+^ T cells and CD20^+^ B cells were detected in RA synovium, whereas OA synovial specimens exhibited only a few scattered T and B cells. IGFBP6 was strongly expressed in different cells of RA synovial lining and sublining layers, including macrophages (type A synoviocytes), fibroblast-like synoviocytes (FLS, type B synoviocytes), microvessels, and infiltrating immune cells (Figure 3E). On the contrary, faint expression of IGFBP6 was detected in cells of OA synovium (Figure 3F). Indeed, densitometric analysis of immunofluorescent staining intensity on ST sections showed that IGFBP6 protein expression was significantly increased either in the lining or in the sublining layers of RA synovium compared with OA (*p* < 0.001 for both) (Figure 3G). To specifically investigate the expression of IGFBP6 in different synovial resident cell types, we carried out double immunofluorescence staining combining anti-IGFBP6 Abs with Abs raised against the pan-endothelial cell marker CD31, the fibroblast marker VIM and the macrophage marker CD68 (Figures 4A–F). As shown in Figures 4A–F, IGFBP6 expression was strongly increased in CD31^+^ vascular endothelial cells, VIM^+^ FLS, and CD68^+^ macrophages of RA ST with respect to OA. Of note, strong IGFBP6 immunopositivity was localized either in the cytoplasm or in the nucleus of RA synovial endothelial cells and FLS as compared with very weak signal detected in the same cell types of OA synovium (Figures 4A–D, insets). As far as infiltrating immune cells are concerned, IGFBP6/CD3 double immunofluorescence staining revealed that the majority of CD3^+^ T cells infiltrating RA synovium displayed very strong immunopositivity for IGFBP6 as compared with OA controls (Figures 5A,B). On the contrary, a few IGFBP6-expressing CD14^+^ monocytes were observed both in RA and OA ST (Figures 5C,D).

### IGFBP6 Induces Immune Cell Migration Which Is Partly Inhibited by dex

**Figure 6:**
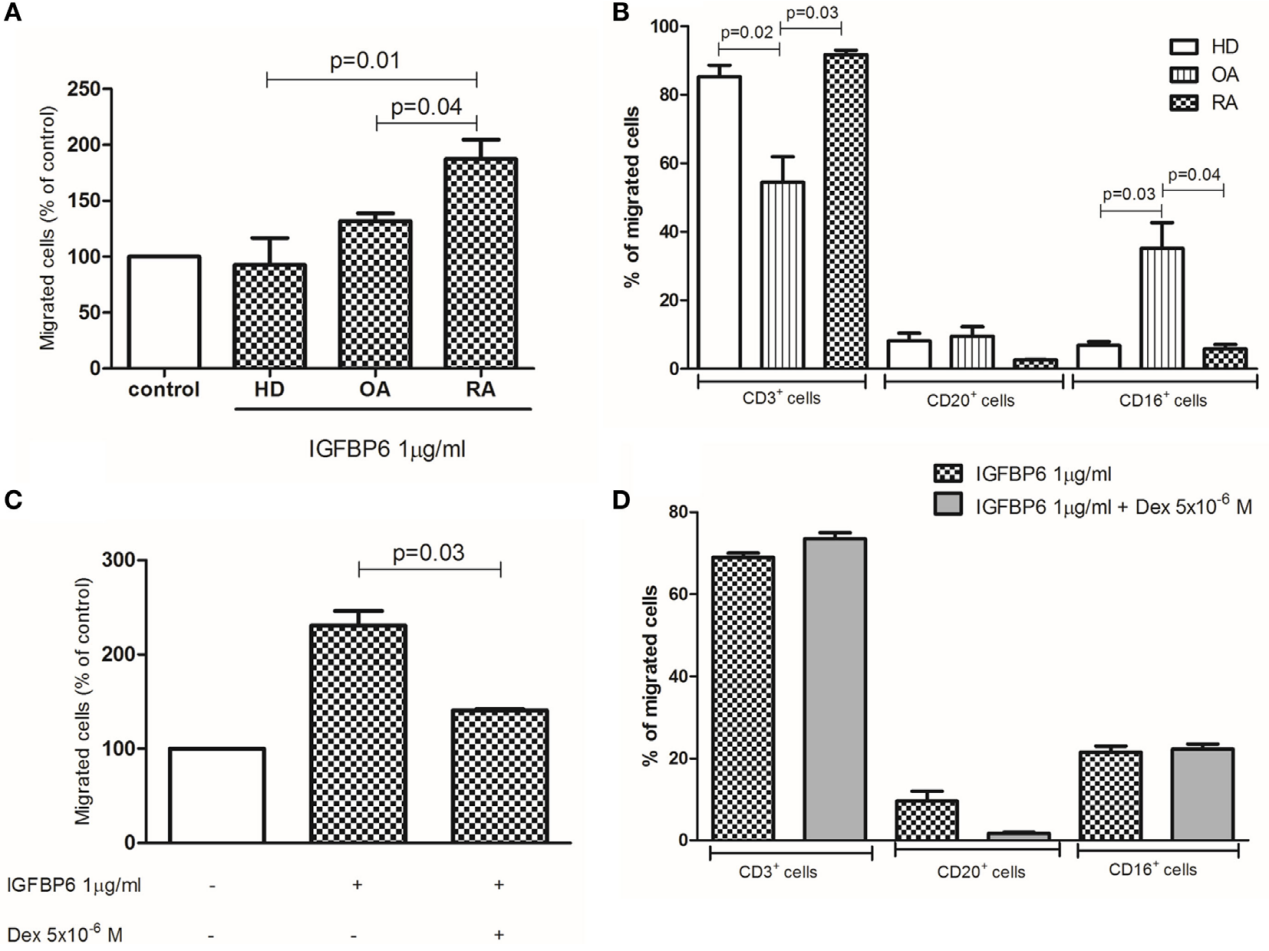
***In vitro* migration assay**. Insulin-like growth factor binding protein 6 (IGFBP6) is able to induce the migration of rheumatoid arthritis (RA) but not healthy donors (HD) and osteoarthritis (OA) peripheral blood mononuclear cells **(A)**. The addition of dexamethasone (dex) in the upper chamber of the transwell partly inhibited such RA cell migration **(B)**. The majority of RA-migrated cells are CD3^+^ T lymphocytes **(C)** and the addition of dex does not affect the proportion of migrated cells **(D)**. All *p* values are calculated with Mann–Whitney *U* test.

Finally, we aimed at demonstrating whether IGFBP6 may act as a chemotactic stimulus for RA immune cells *in vitro*. For this purpose, we set up a migration assay and observed that IGFBP6 was able to induce substantial migration of RA, compared to OA and HD cells (Figure 6A). We then characterized RA-migrated cells by flow cytometry and observed that over 80% of them were CD3^+^ T lymphocytes, while the remaining 20% was a combination of CD20^+^ and CD16^+^ cells (Figure 6B). Furthermore, the addition of dex to the upper chamber was able to partially inhibit the migration of RA cells (Figure 6C). Although partially inhibiting the migration of RA cells, dex did not affect the proportion of migrated cell types being CD3^+^ cells still the majority of them (Figure 6D). Interestingly, the proportion of OA-migrated cells was comparable to that of HD, but with different proportions of cell types in comparison to HD and RA, being the percentage of CD3^+^ cells significantly lower and the percentage of CD16^+^ cells significantly higher. Of interest, all migrated cells coexpressed intracellular IGFBP6.

## Discussion

In recent years, the protein IGFBP6 has been extensively investigated in tumor biology. Although it appears to exert an inhibitory effect on the tumorigenic properties of IGF-II (5, 6), this protein has been raising a growing interest for its properties in promoting cancer cell migration (6–9). On the basis of this observation, we tried to analyze a possible role of IGFBP6 in a chronic inflammatory disorder such as RA, where pannus development and cartilage and bone damage at the joint level recall tumor development and invasion. The results of the present study demonstrated for the first time that the serum levels of this protein are higher in RA than HD. A number of studies have investigated circulating IGFBP6 levels in various neoplastic and non-malignant disorders, including renal, neurologic and ocular diseases, and type 1 diabetes (T1D) in order to evaluate its clinical relevance. However, the majority of these studies gave conflicting data and did not provide significant results from a pathogenic and/or clinical point of view, due to a number of methodological limits (13). Of some interest, studies investigating IGFBP6 in T1D showed higher serum levels of this protein in patients with respect to HD and a direct correlation between higher IGFBP6 levels and diabetic complications (14–16). To note, however, these studies mainly addressed IGF-II-dependent effects of IGFBP6 and speculated that the increase of IGFBP6 in T1D patients could be the consequence of increased IGF-II levels induced by chronic hyperglycemia (16).

It is intriguing to observe that, similar to what observed in the present study, serum IGFBP6 increase was more evident in older patients in several studies. Increased IGFBP6 levels were observed in the serum of aging mice (17) and humans (18) or in senescence induced by doxorubicin in colon cancer (19) or by hydrogen peroxide in fibroblasts (20). It is well known that RA is associated with accelerated senescence of the immune response (21), and, therefore, it is intriguing to speculate that chronic inflammation and disease evolution of RA may represent the stimulus for the progressive increase of IGFBP6 in serum. This hypothesis may be strengthened by the observation that neither different disease activity nor different treatment, but only age, and in consequence disease duration, affected IGFBP6 serum concentration in our study.

The IGF-II/IGFBPs system has been scarcely investigated in RA (11, 22–24). As mentioned above, no data regarding serum IGFBP6 are available in RA. Interestingly, however, IGF-II serum levels were found to be lower in RA than in HD (24). Since IGFBP6 is a direct antagonist of IGF-II, the lower availability of free IGFs in RA serum may be explained by increased binding to circulating IGFBP6 that, according to our data, is enhanced in RA compared to HD. Since the main inflammatory process takes place at the joint level, we sought to evaluate IGFBP6 in SF and ST of RA inflamed joint. Surprisingly, we found that SF IGFBP6 was detectable in RA, but at a lower level compared to OA. On the contrary, this protein was highly expressed in RA, but not OA, ST. Considering the IGFBP6 ability to promote tumor cell migration (6–9), the overexpression of IGFBP6 in ST may suggest its relevance for immune cell migration into inflammatory environment. In order to verify this hypothesis, we performed a series of *in vitro* experiments demonstrating that, in fact, IGFBP6 acts as chemoattractant for RA immune cells, in particular T lymphocytes. Although the low IGFPB6 concentrations in RA SF seem to be apparently in contrast with this idea, our results demonstrated an inverse correlation between these concentrations and WBC count in SF. Since IGFBP6 is internalized by target cells (6) and most of the migrated cells in our *in vitro* system coexpressed IGFBP6, it is intriguing to speculate that the low concentration of IGFBP6 may be due, at least in part, to its internalization by WBC. In addition, since IGFBP6 is susceptible of proteolysis by matrix metalloproteinase 2 (MMP-2) (6) and PLTs are major producer of MMP-2 (25), the inverse relationship between PLT count and IGFBP6 level may also suggest that proteolysis may be another mechanism leading to low IGFBP6 concentrations in RA SF.

The observation that the number of circulating IGFBP6^+^ T cells was significantly lower in RA compared to controls, while numerous IGFBP6-expressing T lymphocytes could be detected in RA ST allows us to speculate that T cells coexpressing IGFBP6 are no longer detectable in RA PB as they are migrated in the synovial membrane. This is supported by the already cited finding showing that the majority of migrated RA T cells coexpress IGFBP6 in our *in vitro* assay. On the other hand, the evidence that circulating IGFBP6^+^CD16^+^ cells were significantly higher in RA PB compared to HD allows us to postulate that monocytes may represent the main source of IGFBP6 to be released and subsequently internalized by T cells.

Besides infiltrating mononuclear cells, other cell types, including FLS, express IGFBP6 in RA ST in our study. In this context, it is important to underline that a recent study demonstrated that IGF-II expression in RA FLS promotes their proliferation, thereby contributing to FLS hyperplasia (26). Moreover, experimental data showed IGFBP6 ability to inhibit fibroblast proliferation by both IGF-dependent and -independent mechanisms (26). The apparent paradox, however, that IGFBP6 was overexpressed in the ST of inflamed joints by several cell types other than infiltrating lymphocytes, may be explained by either IGFBP6 inefficacy in blocking IGF-II or by additional effects exerted by the protein. In this context and according to our data, IGFBP6 was also expressed by synovial microvessels similar to other molecules that orchestrate the leukocyte-adhesion cascade. Since IGFBP6 can exert an inhibitory role on angiogenesis in neoplastic disorders (27), we cannot rule out that the expression of this protein on microvessels may be due, at least in part, by IGFBP6 activity to limit RA neoangiogenesis. However, on the basis of the present demonstration that IGFBP6 is chemotactic for RA T lymphocytes *in vitro* and that the majority of T cells infiltrating RA synovium displayed very strong immunopositivity for IGFBP6, it is tempting to speculate that the main pathogenic roles exerted by IGFBP6 in RA may be that of T-cell chemoattractant from bloodstream to synovial membrane.

In conclusion, our data highlight the potential roles of IGFBP6 in RA immune cells *in vitro*. Among these effects, this protein appears to put forward as a novel chemotactic agent driving T-cell migration from PB to the inflamed joints in RA and, in consequence, as new potential therapeutic target in this disorder. This finding may be of great value, since the interference with immune cell migration for therapeutic purposes in RA has been extensively pursued, but the blockade of chemokines has not provided consistent benefit up to now (3, 4). In this context, it is of interest our results showing that corticosteroids are able, at least in part, to prevent IGFBP6-induced chemotaxis, probably through a non-genomic effect considered the celerity of action in our *in vitro* assays (28). Further studies are in progress, therefore, to better clarify both pathogenic and therapeutic implications of these findings.

## Ethics Statement

This study was carried out in accordance with the recommendations of the Comitato Etico delle Aziende Sanitarie dell’Umbria with written informed consent from all subjects. All subjects gave written informed consent in accordance with the Declaration of Helsinki. The protocol was approved by the Comitato Etico delle Aziende Sanitarie dell’Umbria.

## Author Contributions

AA and OB concepted and designed the study, performed experiments and data analysis, arranged the images and drafted the manuscript. MM designed and performed experiments, performed data analysis, arranged the images and drafted the manuscript. GC, VV, and EB recruited patients and controls, collected clinical data, and critically revised the manuscript for important intellectual content. RG and AL participated to study design and coordination and critically revised the manuscript for important intellectual content. All authors approved the final version of the manuscript.

## Conflict of Interest Statement

All the authors disclose any financial support or other benefits from commercial sources for the work reported in this paper, or any other financial and non-financial interests that any of them may have, which could create a potential conflict of interest or the appearance of a conflict of interest with regard to the work. The reviewer, AC, and handling editor declared their shared affiliation, and the handling editor states that the process nevertheless met the standards of a fair and objective review.
